# Breast Cancer Subtypes Based on Hypoxia-Related Gene Sets Identify Potential Therapeutic Agents

**DOI:** 10.3389/fmolb.2022.900005

**Published:** 2022-06-29

**Authors:** Zhenchong Xiong, Weiling Huang, Wenjing Zhong, Jianchang Fu, Jikun Feng, Xi Wang, Feihai Ling

**Affiliations:** ^1^ State Key Laboratory of Oncology in South China, Collaborative Innovation Center of Cancer Medicine, Department of Breast Oncology, Sun Yat-sen University Cancer Center, Guangzhou, China; ^2^ State Key Laboratory of Oncology in South China, Collaborative Innovation Center of Cancer Medicine, Department of Pathology, Sun Yat-sen University Cancer Center, Guangzhou, China; ^3^ Department of Breast Surgery, Zhongshan City People’s Hospital, ZhongShan, China

**Keywords:** breast cancer, hypoxia-related gene sets, clustering analysis, therapeutic agent, subtypes

## Abstract

**Purpose:** The hypoxic tumor microenvironment was reported to be involved in different tumorigenesis mechanisms of breast cancer (BC). We aimed to establish a hypoxia-related gene signature to identify a new BC subtype through the clustering analysis and explore potential compounds targeting the BC subtypes.

**Methods:** Gene expression data and clinical features of BC and adjacent non-tumor tissues were downloaded from the Cancer Genome Atlas-Breast cancer (TCGA-BRCA) database. We comprehensively revealed the activity changes of Gene Ontology (GO) biological processes (BP) gene sets in BC by gene set variation analysis (GSVA) and identified three hypoxia-related BC subtypes. We then matched the differentially expressed gene profile of each subtype with the gene profile in CMap database to identify the potential agents targeting the BC subtypes.

**Results:** 562 of Gene Ontology biological processes gene sets significantly correlated with hypoxia score in breast cancer. 969 BC patients were clustered into three subtypes based on the enrichment score of hypoxia-associated gene sets. Subtype 1 patients displayed better survival than subtype 2 and 3. KEGG pathway enrichment analysis of each subtype was performed based on the unique differential expression genes profile. In subtype 1, the upregulated genes were associated with lipid and amino acid metabolism regulation; in subtype 2, the upregulated genes were associated with metabolic energy regulation, while in subtype 3, the upregulated genes were associated with apoptosis and protein process. Using the CMap database, 55, 111 and 63 compounds were identified, targeting subtype 1, 2, and 3, respectively.

**Conclusion:** In this study, novel hypoxia-related subtypes were developed for patients with BC. In addition, biological processes associated with differential expression genes profile and potential therapeutic target compounds were identified in each subtype. The new classification might provide a better understanding of the role of hypoxia in breast cancer and more individualized treatment for patients.

## Introduction

More than 2,200,000 women were diagnosed with breast cancer (BC) in 2021, making BC the most commonly diagnosed cancer among women and the leading cause of cancer death for women worldwide (Sung et al., 2021). BC is a heterogeneous disease with respect to molecular alterations, cellular composition, and clinical outcome. Based on the intrinsic molecular subtypes, defined by mRNA expression of 50 genes (PAM50), breast cancer is divided into luminal A, luminal B, HER2 (human epidermal growth factor receptor 2)-enriched, basal-like, and normal-like (Parker et al., 2009). Additionally, gene expression profiling by microarray such as 21-gene recurrence score assay (Oncotype DX) (Sparano et al., 2018) and the 70-gene MammaPrint (Cardoso et al., 2016) microarray can be used to provide prognostic and predictive information beyond standard clinical assessment. However, some patients still have tumor progression due to the lack of suitable therapeutic agents or treatment resistance.

Hypoxia is one of the hallmarks of cancer (Gilkes et al., 2014; de Heer et al., 2020). Low intratumoral O2 levels (hypoxia) are associated with angiogenesis, metabolic reprogramming, extracellular matrix remodeling, epithelial–mesenchymal transition, motility, invasion, metastasis, cancer stem cell maintenance, immune evasion, and chemo-resistance and radiation therapy (Schito and Semenza, 2016). Hypoxia leads to increased activity of hypoxia-inducible factors (HIFs). HIF-1 promotes the expression of hundreds of genes involved in cell autonomous and non-autonomous adaptations to hypoxia. On the one hand, HIF-α can be upregulated at the protein level *via* mTOR or the mRNA level *via* STAT3 and NF-κB signaling (Karar and Maity, 2011). Also, HIF-1 promotes lymphatic metastasis of breast cancer by direct transactivation of the gene encoding platelet-derived growth factor B (PDGF-B), which has proliferative and chemotactic effects on lymphatic endothelial cells (Schito et al., 2012). The newly formed vasculature is disorganized and leaky, which facilitates tumor cell invasion and metastasis, impairs drug delivery, and further aggravates hypoxia in the tumor and the microenvironment (Martin et al., 2019). On the other hand, in cancer-associated fibroblasts (CAFs), HIF-α mediates extracellular matrix (ECM) remodeling, in which metabolic reprogramming supporting cell survival (Gilkes et al., 2014). In addition, HIF-α promotes the expression of cytokines that suppress the adaptive immune system by stimulating the recruitment and activation of myeloid-derived suppressor cells (MDSCs), regulatory T cells (Treg) and tumor associated macrophages (TAMs) leading to an immunosuppressive environment (Palazón et al., 2012). The study of tumor genetic changes in the hypoxia environment might provide hints for cancer treatment (Cosse and Michiels, 2008).

Therefore, it is necessary to deepen the understanding of the heterogeneity of breast cancer and explore hypoxia-related subtypes and therapeutic agents to provide individualized treatment for patients. In the present study, by performing a comprehensive bio-informatics analysis based on the Cancer Genome Atlas-Breast cancer (TCGA-BRCA) datasets, we aimed to establish a hypoxia-related gene signature to identify a new BC subtype through the clustering analysis and explore potential compounds targeting the BC subtypes.

## Materials and Methods

### Data Collection and Processing

First, gene expression data and clinical features of BC and adjacent non-tumor tissues were downloaded from the Cancer Genome Atlas-Breast cancer (TCGA-BRCA) database (https://portal.gdc.cancer.gov/). Second, gene expression data and clinical features of BC tissues were downloaded from the METABRIC database (https://www.nature.com/articles/nature10983). Third, the hypoxia score (Buffa, Ragnum, and Winter) of breast cancer tissues were obtained from cbioportal (http://www.cbioportal.org/). Finally, the Gene Ontology (GO) biological processes (BP) gene sets were downloaded from Gene Set Enrichment Analysis (GSEA) (http://www.gsea-msigdb.org/gsea/index.jsp).

### Gene Set Variation Analysis

GSVA was performed to quantify the relative enrichment of gene sets in BC and adjacent non-tumor tissues, which are able to reveal the activity variation of a set of genes involved in the particular biological processes (Hänzelmann et al., 2013). GSVA was performed by R package ‘GSVA’ (Hänzelmann et al., 2013).

Hypoxia scores were calculated for all TCGA-BRCA tumors with mRNA expression data using mRNA-expression-based signatures of tumor-hypoxia developed by Winter et al. (2007), Buffa et al. (2010), and Ragnum et al. (2015). The hypoxia score (Buffa, Ragnum, and Winter) was public data provided in cBioPortal (http://www.cbioportal.org/study/summary?id=brca_tcga). The enrichment score of gene sets and the hypoxia score (Buffa, Ragnum, and Winter) were used to identify the hypoxia-associated BP gene sets. The correlation between the enrichment score of BP gene sets and hypoxia score was analyzed through the spearman correlation analysis. Gene sets with the Spearman coefficient >0.3 or < −0.3 (***p*** < 0.05) were defined as the hypoxia-associated BP gene sets.

### Identification of Hypoxia-Related Breast Cancer Subtype Through the Clustering Analysis

Enrichment score of hypoxia-related BP gene sets in breast cancer tissues were calculated with GSVA. Cox regression model was used to evaluate the survival correlation of enrichment score of hypoxia-related BP gene sets. Gene sets with enrichment score significantly correlated with patients’ survival were further included in the cluster analysis. The optimal number of clusters (K) was generated by R package ‘factoextra’ (Garcia-Rudolph et al., 2020). Consensus Clustering analysis was performed to distinguish the molecular subtype of BC based on the Gene sets enrichment score by R package “CancerSubtypes” (Xu et al., 2017). Silhouette width ranging from −1 to 1 was used to measure the accordance of the subtype clustering (the value of silhouette width being close to 1 means that a sample is well matched to its identified subtype compared to other subtypes, and vice versa).

### Identification of Potential Compounds Targeting the Breast Cancer Subtypes

The recently updated Connectivity Map (CMap) database is a platform for discovering connections between gene profile, drugs sensitivity, and diseases states (Subramanian et al., 2017). The CMap data and tools are available on https://clue.io. The differential expression genes (DEGs) between each subtype of BC tissues and adjacent non-tumor tissues was identified using R package “limma” (Ritchie et al., 2015). After the exclusion the repeating DEGs, the unique up/down-regulated gene profile was obtained, and the top 300 genes (150 upregulated and 150 downregulated) were inputted to the CMap database. Compounds with an enrichment score ≤ −90 in BC cell lines were selected as potential therapeutic methods for each subtype of BC.

### Statistical Analysis

The statistical analyses in this study were performed with R software. A *p*-value of <0.05 was considered statistically significant. The Kyoto Encyclopedia of Genes and Genomes (KEGG) pathway enrichment analysis was performed by R package “clusterProfiler” (Yu et al., 2012). The Kaplan-Meier survival curve and log-rank test were used to analyze overall survival (OS) between different groups of BC patients.

## Results

### Identification of Hypoxia-Associated Biological Processes Gene Sets and Hypoxia-Related Breast Cancer Subtypes.

**FIGURE 1:**
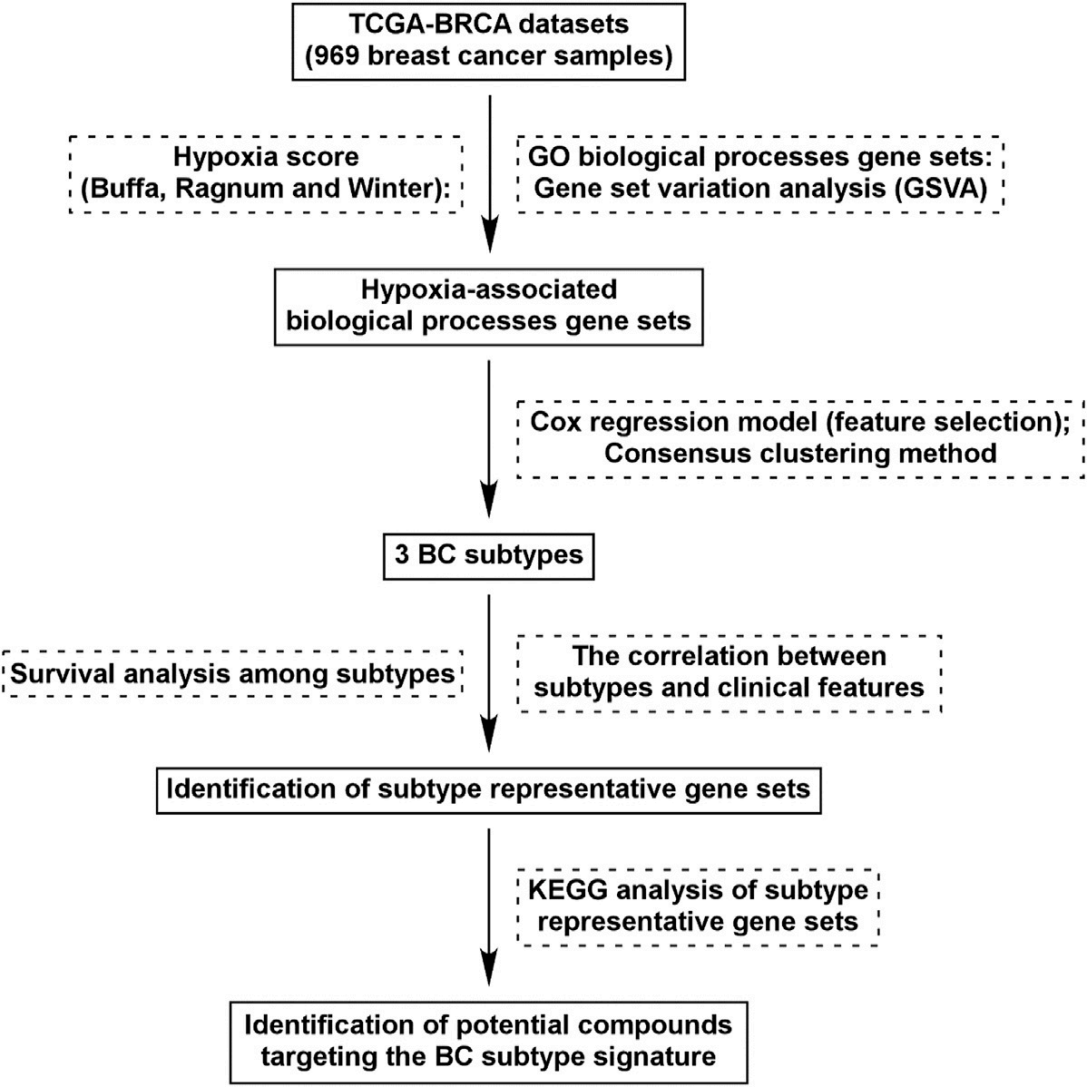
Flow chart of study design. We identified 562 GO BP gene sets which were commonly correlated with hypoxia score (Buffa, Ragnum, and Winter) in BC tissues. Using Cox regression model and Consensus clustering method, three subtypes were identified. Survival analysis and correlation analysis between subtypes and clinical features were performed. Through analysis of differential gene expression, genes uniquely up/down-regulated in each subtypes were identified and KEGG analysis was performed. Identification of potential compounds of each subtype was performed using the unique gene profile.

A total of 7481 GO BP gene sets (c5.go.bp.v7.4.symbols) were obtained from GSEA, and 2474 gene sets were excluded due to the missing expression of gene in BC tissues (Figure 1). Therefore, 5007 GO BP gene sets were included to identify hypoxia-associated BP gene sets, and 562/5007 gene sets were commonly correlated with hypoxia score (Supplementary Table S1).

**FIGURE 2:**
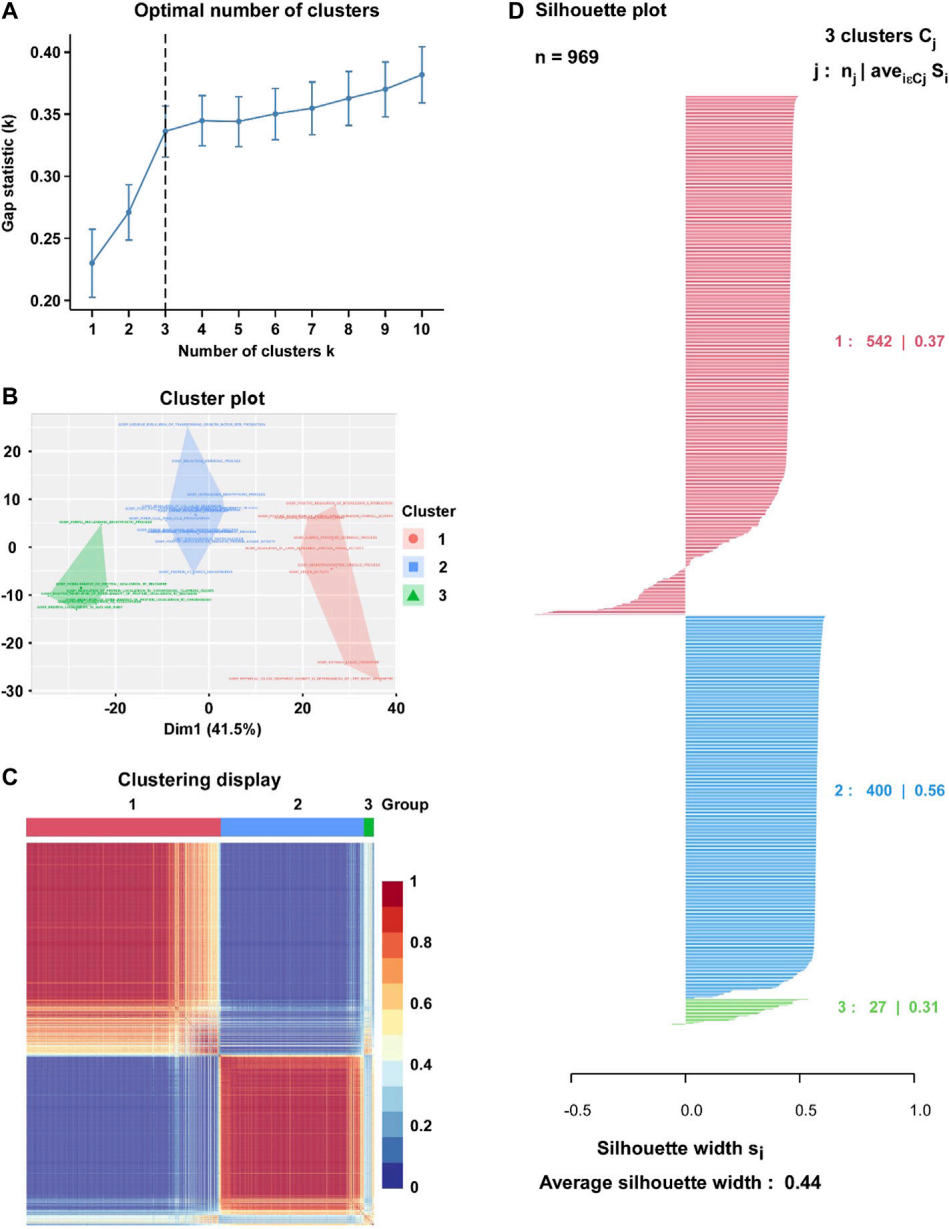
Identification of hypoxia-related BC subtype through the Clustering analysis. **(A)** Factoextra package was used to calculate the optimal number of clusters (K = 3). **(B)** Visualize clusters using factoextra. **(C)** Heatmap of the sample similarity matrix based on the cluster results. **(D)** Silhouette width plots for the identified cancer subtypes: the value of silhouette width being close to 1 means that a sample is well matched to its identified subtype compared to other subtypes, and vice versa.

Based on the enrichment score of 562 hypoxia-associated gene sets, we purposed to divide BC patients into different subtypes. The factoextra package was used to calculated the optimal number of clusters (K = 3) to optimize the cluster analysis (Figure 2A). Then, Cox regression model was used for the feature selection, and 31 gene sets were eventually determined for the cluster analysis (Figure 2B). By Consensus Clustering method, 969 BC patients were clustered into three subtypes (subtype 1: *n* = 542; subtype 2: *n* = 400; subtype 3: *n* = 27) (Figures 2C,D). The silhouette width plots showed that the average silhouette width was 0.44, indicating that the samples are well matched to their identified subtype compared to other subtypes (Figure 2D).

### The Correlation Between Hypoxia-Related Breast Cancer Subtypes and Clinical Features.

Among three subtypes, subtype 1 BC tissues exhibited significantly lower hypoxia scores than subtypes 2 and 3 (Supplementary Figure S1A). In addition, a significantly higher proportion of patients in subtype 1 were diagnosed with hormone receptor positive (HR +) BC, while patients in subtype 2 and subtype 3 were more likely to be TNBC and Her-2 +, respectively (Table 1). The correlation between clinical characteristics and subtypes is presented in Table 1.

**TABLE 1:** The correlation between clinical characteristics and subtypes.

	Subtype 1 (*n* = 542)	Subtype 2 (*n* = 400)	Subtype 3 (*n* = 27)	*p value*
T stage (%)				0.01
T1	171 (31.5%)	82 (20.5%)	8 (29.6%)	
T2	279 (51.5%)	254 (63.5%)	15 (55.6%)	
T3	73 (13.5%)	48 (12%)	3 (11.1%)	
T4	18 (3.3%)	14 (3.5%)	1 (3.7%)	
TX	1 (0.2%)	2 (0.5%)	0 (0%)	
N stage (%)				0.444
N0	256 (47.2%)	191 (47.7%)	11 (40.7%)	
N1	185 (34.1%)	131 (32.8%)	11 (40.7%)	
N2	48 (8.9%)	50 (12.5%)	3 (11.2%)	
N3	44 (8.1%)	20 (5%)	2 (7.4%)	
NX	9 (1.7%)	8 (2%)	0	
M stage (%)				0.064
M0	440 (81.2%)	342 (85.4%)	22 (81.5%)	
M1	7 (1.3%)	11 (2.8%)	0 (0%)	
MX	95 (17.5%)	47 (11.8%)	5 (18.5%)	
Molecular subtype (%)				<0.001
Luminal A/B	473 (87.3%)	239 (59.8%)	20 (74.1%)	
Her-2	9 (1.7%)	24 (6%)	4 (14.8%)	
TNBC	36 (6.6%)	105 (26.2%)	2 (7.4%)	
Unknown	24 (4.4%)	32 (8%)	1 (3.7%)	
Vital status (%)				0.007
Alive	479 (88.4%)	327 (81.7%)	21 (77.8%)	
Death	63 (11.6%)	73 (18.3%)	6 (22.2%)	
Median survival time (Months, 95%CI)	219.8 (90.1–349.5)	115.7 (97.3–134.1)	75.8 (51.6–100)	

*p* value were calculated by Fisher’ exact test. TNBC, triple negative breast cancer; CI, confidence interval.

**FIGURE 3:**
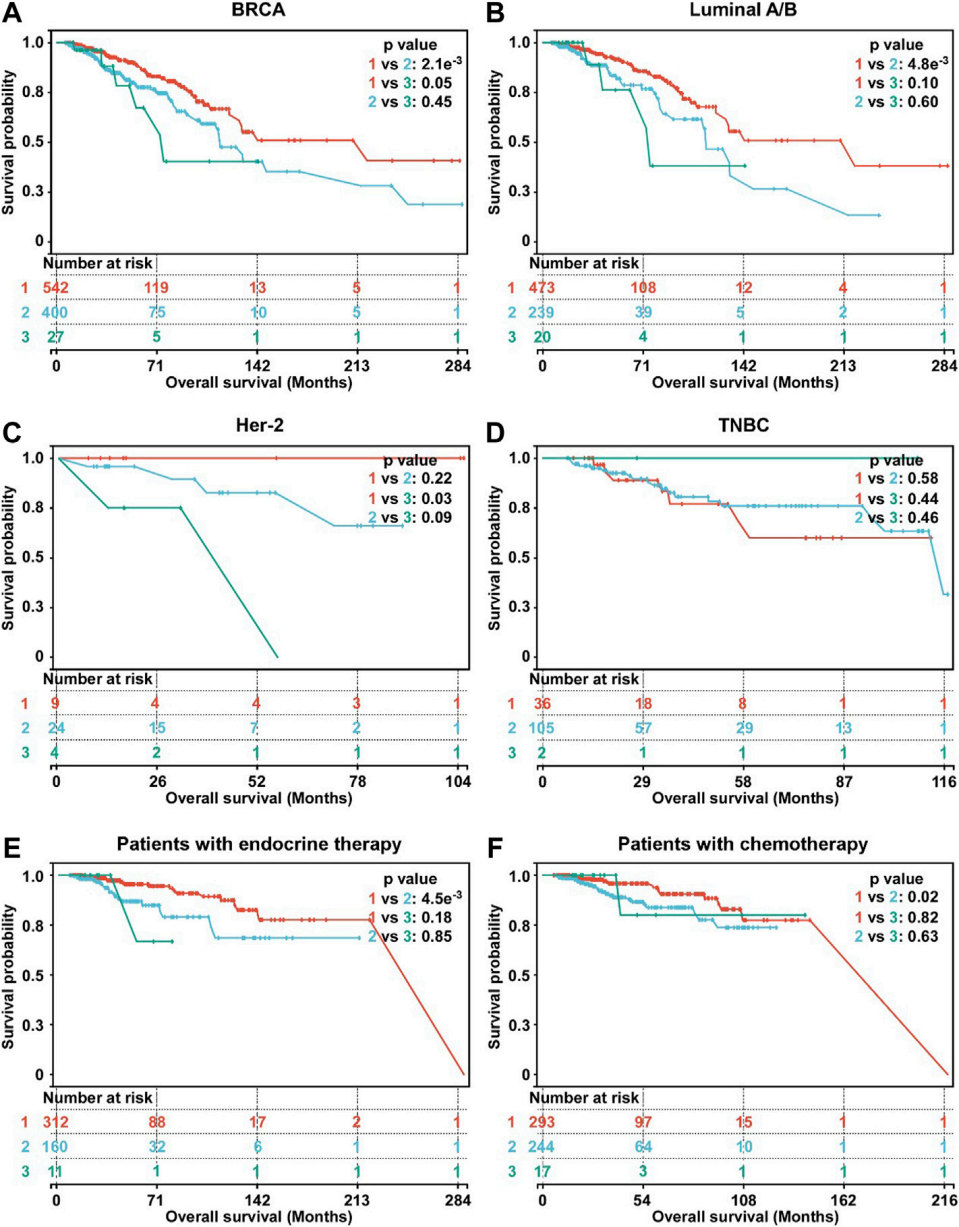
Survival analysis of hypoxia-related BC subtypes. **(A)** Survival analysis of hypoxia-related BC subtypes in BC patients (*n* = 969). **(B)** Survival analysis of hypoxia-related BC subtypes in luminal A/B BC patients (*n* = 732). **(C)** Survival analysis of hypoxia-related BC subtypes in Her-2 BC patients (*n* = 37). **(D)** Survival analysis of hypoxia-related BC subtypes in TNBC patients (*n* = 143). **(E)** Survival analysis of hypoxia-related BC subtypes in BC patients with endocrine therapy (*n* = 483). **(F)** Survival analysis of hypoxia-related BC subtypes in BC patients with chemotherapy (*n* = 554). For A-F, *p*-values were determined by log-rank test.

The Kaplan-Meier survival was used to evaluate the association between OS and hypoxia-associated subtypes. In BC, patients in subtype 1 exhibiting a lower hypoxia score displayed a better survival than subtype 2 and 3 (Figure 3A). OS of patients in subtype 1 was better than OS of subtype 2 in HR + BC (luminal A/B) and OS of subtype 3 in Her-2 BC (Figures 3B,C). In TNBC, no OS difference was observed among three subtypes (Figure 3D). Moreover, subtype 1 BC patients displayed better OS than subtype 2 BC patients in patients with either endocrine therapy or chemotherapy (Figures 3E,F). Overall, subtype 1 BC patients displayed a better outcome than the other two subtypes. Moreover, we inrolled data of BC tissues from the METABRIC database. A total of 1897 BC patients in METABRIC cohort were similarly classified into three subtypes using the clustering analysis based on the enrichment score of hypoxia-associated gene sets. Next, we evaluated the association between the hypoxia subtypes and patients OS. Consistent with our previous findings, subtypes 1 patients exhibited better survival than patients with subtype 2 or 3 (Supplementary Figure S3). In patients with endocrine therapy or chemotherapy, subtype 1 patients had longer OS than those of subtype 2 (Supplementary Figures S3E,F).

### Identification of Unique Differential Expression Gene Profile in Hypoxia-Related Breast Cancer Subtypes

**FIGURE 4:**
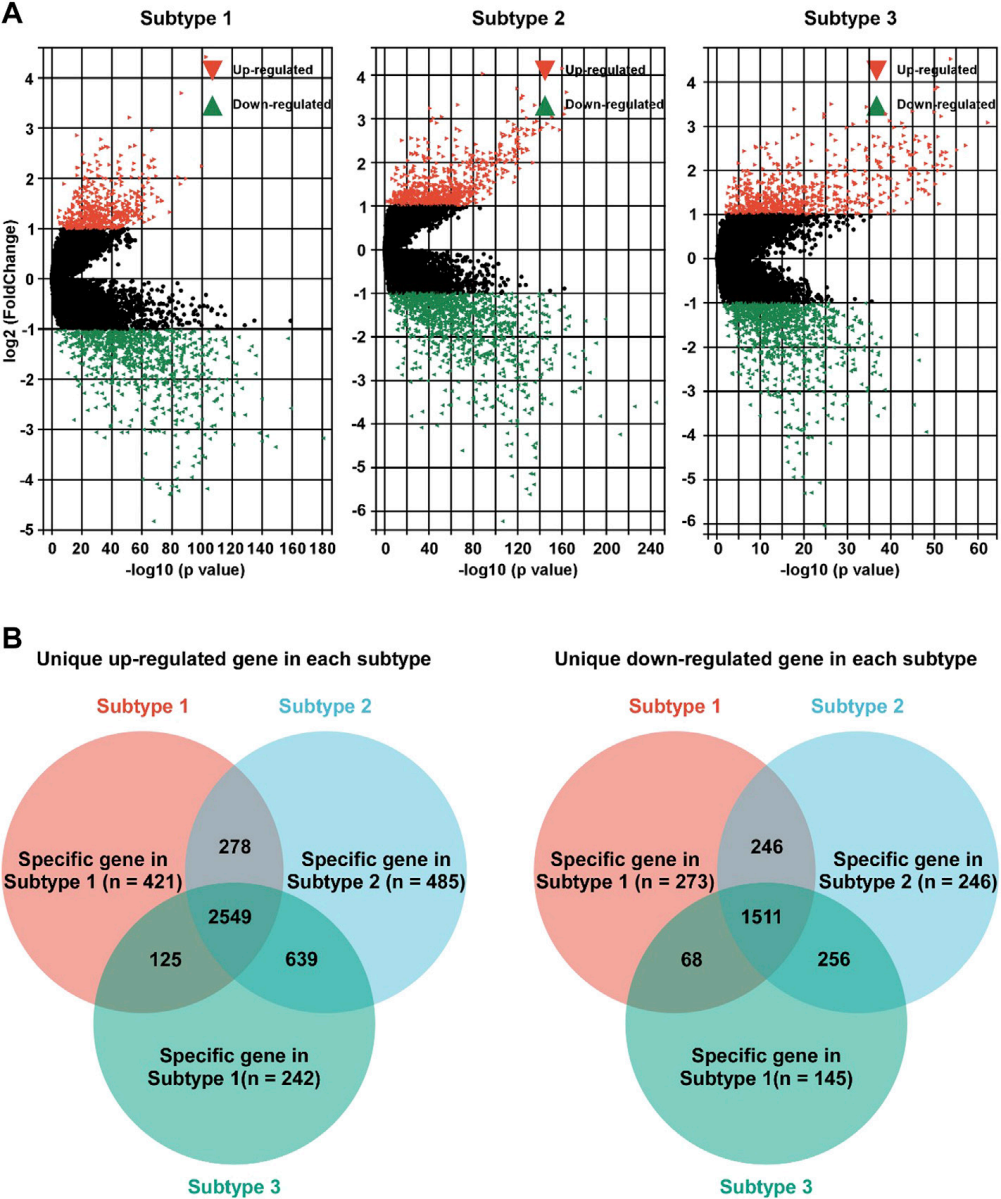
Identification of unique DEG profile in hypoxia-related BC subtypes. **(A)**. Volcano plot of the DEGs between adjacent non-tumor tissues and subtype 1 (left panel), subtype 2 (middle panel), or subtype 3 (right panel). **(B)** Unique up-regulated (left panel)/down-regulated (right panel) DEG profile of each hypoxia-related BC subtypes were identified.

To further study the characteristics of each subtype, DEG profile of each subtype was identified (Figure 4A). In subtype 1 BC, 421 and 273 gens were uniquely up/down regulated, respectively (Figure 4B). In subtype 2 BC, 485 were uniquely up regulated, and 246 gens were down regulated (Figure 4B). In subtype 3 BC, 242 gens were uniquely up regulated, and 145 were down regulated (Figure 4B).

**FIGURE 5:**
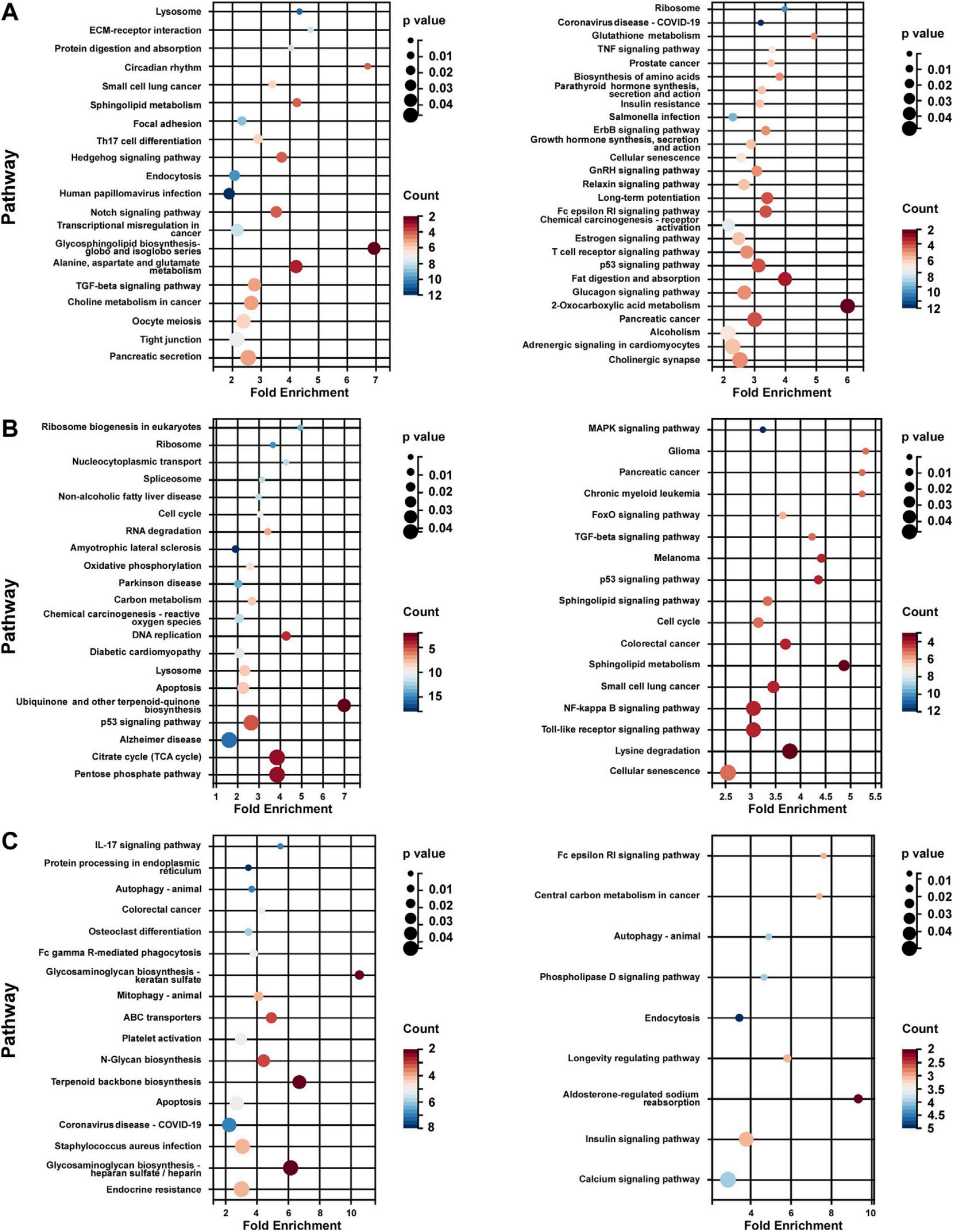
KEGG analysis of unique DEG profile in hypoxia-related BC subtypes. **(A)** KEGG analysis of uniquely up-regulated (left panel)/down-regulated (right panel) DEG profile in hypoxia-related BC subtype 1. **(B)** KEGG analysis of uniquely up-regulated (left panel)/down-regulated (right panel) DEG profile in hypoxia-related BC subtype 2. **(C)** KEGG analysis of uniquely up-regulated (left panel)/down-regulated (right panel) DEG profile in hypoxia-related BC subtype 2. For **(A–C)**, Fold Enrichment were calculated as GeneRatio/BgRatio.

KEGG pathway enrichment analysis of each subtype was performed based on the unique DEG profile. In subtype 1, the upregulated genes were associated with lipid and amino acid metabolism regulation (sphingolipid, glycosphingolipid, choline, alanine, aspartate and glutamate), Notch pathway, and TGF-beta pathway; the down-regulated genes were associated with growth hormone signaling (Erbb pathway, growth hormone synthesis), estrogen pathway, and glycol metabolism (insulin resistance, glucagon signaling) (Figure 5A). In subtype 2, the up-regulated genes associated with metabolic energy regulation (carbon metabolism, reactive oxygen species (ROS) and citrate cycle), while the down-regulated genes associated with FoxO pathway, MAPK pathway, and sphingolipid pathway (sphingolipid signaling and sphingolipid metabolism) and so on (Figure 5B). In subtype 3, the up-regulated genes associated with apoptosis and protein process (protein processing in endoplasmic reticulum, N-Glycan biosynthesis) (Figure 5C).

### Identification of Potential Compounds Targeting the Breast Cancer Subtype

**FIGURE 6:**
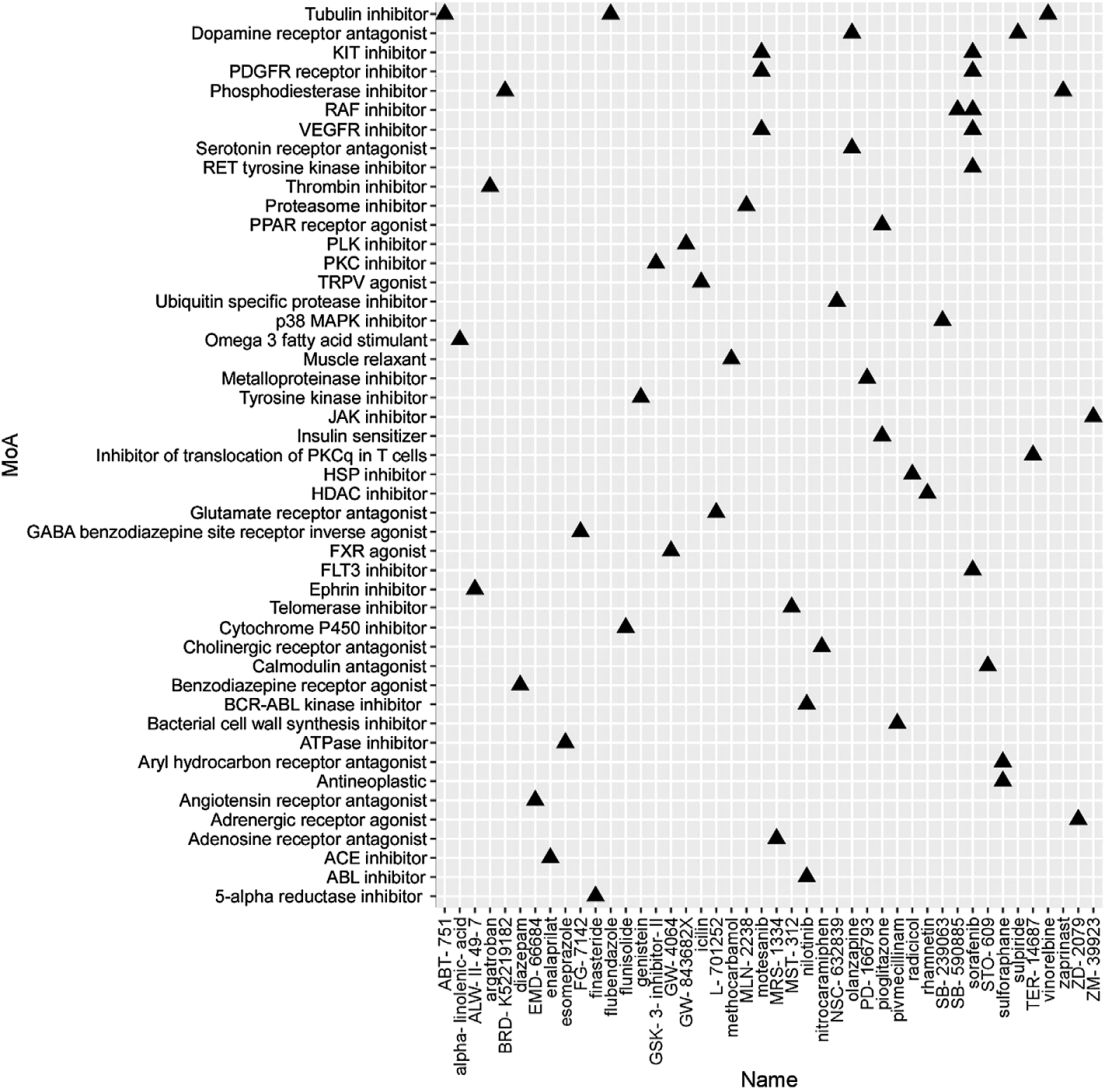
Heatmap of potential therapeutic compounds for subtype 1 and the relative MoA. The above compounds have an enrichment score < −90 in BC cell line and might be able to target the unique gene profile of subtype 1.

**FIGURE 7:**
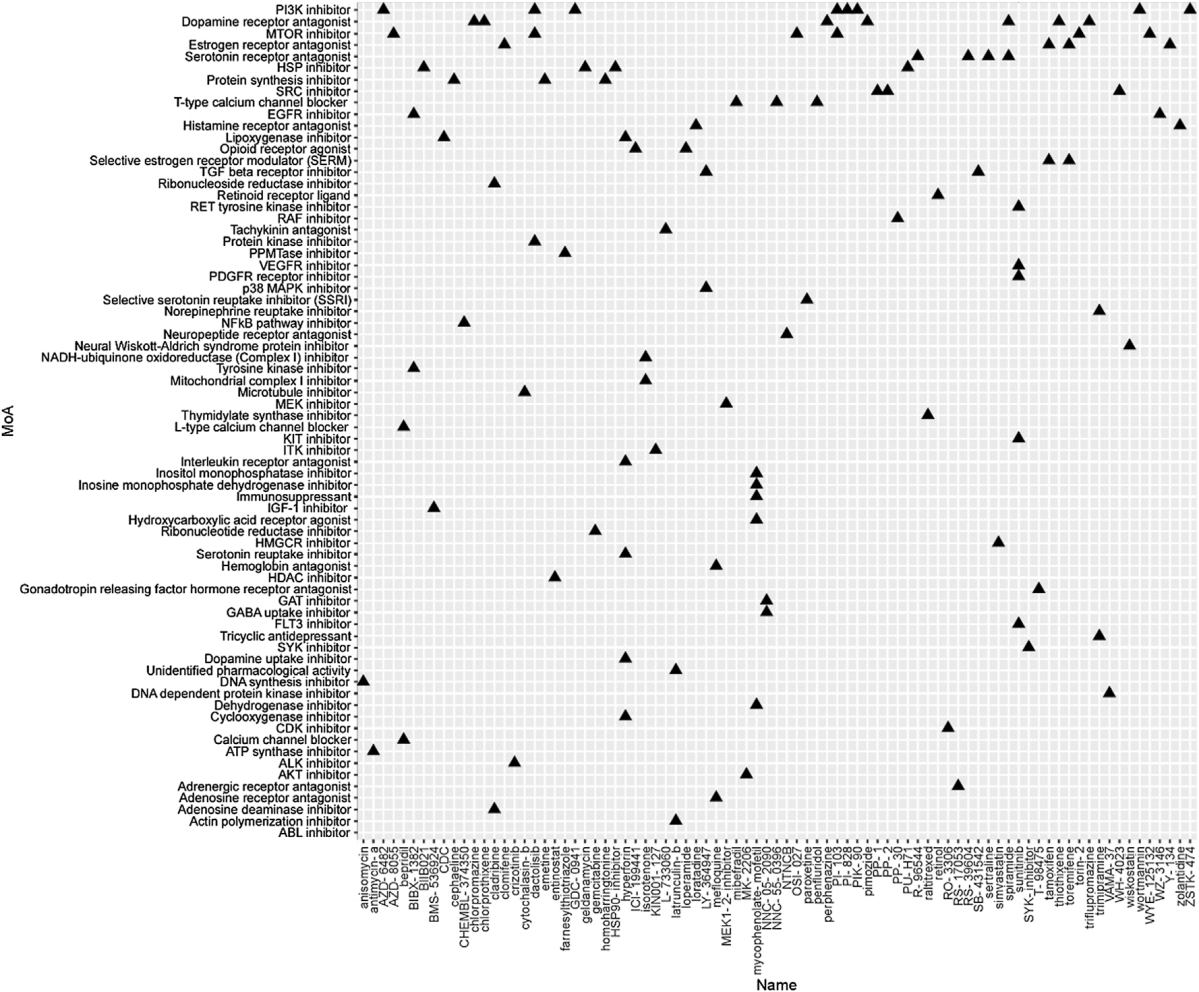
Heatmap of potential therapeutic compounds for subtype 2 and the relative MoA. The above compounds have an enrichment score < −90 in BC cell line and might be able to target the unique gene profile of subtype 2.

**FIGURE 8:**
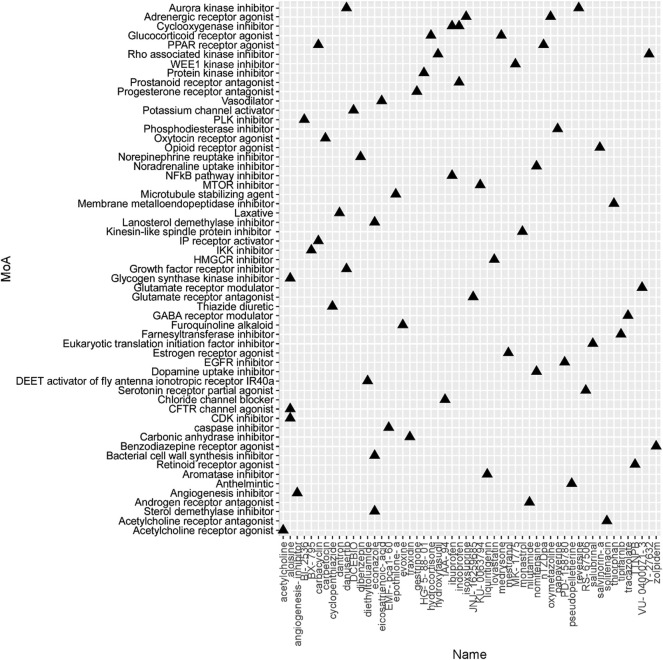
Heatmap of potential therapeutic compounds for subtype 3 and the relative MoA. The above compounds have an enrichment score < −90 in BC cell line and might be able to target the unique gene profile of subtype 3.

To identify the potential agents targeting the BC subtypes, we matched the DEG profile of each subtype with the gene profile in CMap database. Compounds with enrichment score < −90 were negatively correlated with the input gene profiles, indicating the therapeutic potential. 55, 111, and 63 compounds were identified targeting subtypes 1, 2 and 3, respectively (Figures 6–8). 55 compounds referring 47 mechanisms of action (MoA) were identified targeting subtype 1 (Figure 6). 111 compounds referring 72 mechanisms of action (MoA) were identified targeting subtype 2 (Figure 7). 63 compounds referring 57 mechanisms of action (MoA) were identified targeting subtype 3 (Figure 7).

## Discussion

Hypoxia, a hallmark of tumor, was caused by rapid proliferation of tumor cells and the intercapillary distance longer than that of oxygen diffusion (Gilkes et al., 2014). Hypoxia-associated genes (such as HIFs, ARD1A, FIH) and their target gene products are known to be hyperactivated in tumor, which involved in different tumoral mechanisms of cancer. Previous studies have addressed the vital roles hypoxia status plays in the failure of conventional cancer therapies and poor prognosis of multiple cancer such as liver cancer (Bao and Wong, 2021), bladder cancer (Zhang et al., 2021), glioblastoma (Wang et al., 2020) and breast cancer (McAleese et al., 2021). Therefore, Hypoxia-associated genes can be widely used as promising prognostic predictors and therapeutic targets for breast cancer. In the present study, we identified hypoxia-associated BP gene sets and hypoxia-related BC subtypes and explored potential compounds targeting the BC subtype, which might be helpful to increase our knowledge on hypoxia-related phenotypes and associated potential therapeutic targets in breast cancer.

It has been reported that metabolism reprogramming is indispensable for the adaptation of the hypoxia environment in breast cancer (Tang et al., 2021). Moreover, heterogeneity was observed in breast cancer patients regarding metabolic changes (Gong et al., 2021). Each subtype has a distinct proliferation rate, metastatic capacity, and metabolic phenotype and genotype. For instance, previous study showed that various phospholipidsand sphingolipids are upregulated in ER-subtypes relative to ER+ (He et al., 2015). A main regulator of glutamine-related metabolic rewiring, MYC, facilitates excess glutamine uptake by inducing the expression of glutamine transporters and glutaminemetabolizing enzymes in breast cancers (Yue et al., 2017). This molecular mechanism is upregulated in the luminal B, TNBC, and HER2+ subtypes rather than luminal A subtypes (Craze et al., 2018). In our study, although a significantly higher proportion of patients in subtype 1 were diagnosed with hormone receptor positive (HR +) BC, the up-regulated genes associated with lipid and amino acid metabolism regulation (sphingolipid, glycosphingolipid, choline, alanine, aspartate and glutamate) in this subtype. In hypoxia environment, glutamine metabolism plays an important role in cancer progression. Morotti et al. show that hypoxia induces SNAT2, an glutamine transporter, which causes resistance to antihormone therapy. Hypoxia-inducible factor 1α compensates for the loss of expression of estrogen receptor-α (ERα) for maintaining SNAT2 expression under hypoxia or endocrine therapies. SNAT2 overexpression produces complete resistance to antiestrogen therapy *in vivo* and is induced in tamoxifen resistance, and its expression is associated with poor survival in breast cancer and resistance to endocrine therapy in ERα+ luminal B patients (Morotti et al., 2019). The metabolic relationship between the existing molecular subtypes and our proposed hypoxia-related BC subtypes is expected to provide a new idea for the individual therapy of breast cancer.

In addition, patients in subtype 2 and 3 were more likely to be TNBC and Her-2+ and had worse outcome than patients in subtype1. In subtype 2, the upregulated genes were associated with metabolic energy regulation [carbon metabolism, reactive oxygen species (ROS), and tricarboxylic acid cycle (TRCs)]. In subtype 3, the upregulated genes were associated with apoptosis, and protein process (protein processing in endoplasmic reticulum, N-Glycan biosynthesis). Similar to the present study, preclinical studies suggest that TNBC relies more on the glucose metabolism. Transporters involved in macronutrient uptake and metabolic enzymes, such as GLUT1, SLC1A5, SLC7A5, GLS1, and PGDH, are upregulated in TNBC (Budczies et al., 2013; Kulkoyluoglu-Cotul et al., 2019). MYC mentioned above also upregulates serine, glycine, and tryptophan uptake and the synthesis of one-carbon units, resulting in a more active TCA cycle in HER2+ and TNBC breast cancer subtypes (Kim et al., 2013). Showed that HIF1a promotes tumor growth and metastasis by promoting anaerobic glycolysis and lactic acid production in a hypoxic environment Semenza (2013). Pyruvate dehydrogenase kinase (PDK) is a HIF-induced key regulator of lactate production *via* inhibition of pyruvate dehydrogenase (PDH), which rapidly inhibits the first step of the Krebs cycle during hypoxia (Kim et al., 2006). HIF1α induces glucose transporter (GLUT) expression for uptake of extracellular glucose and increases glycogen synthesis and breakdown as an additional glucose source to sustain glycolytic and pentose phosphate flux. Besides, glycogen metabolism has been implicated in improved ROS scavenging, survival after reoxygenation, cell migration, and radioresistance in BC (Altemus et al., 2019). ROS, produced due to dysfunction of the mitochondrial electron transport chain under hypoxic or hyperoxic conditions, was the prime cause of tumor cell death (Xiang and Semenza, 2019). Show that hypoxia promotes the growth of BCs through the actication of the GSH-ROS pathway Tang et al. (2019). In conclusion, our study showed that different hypoxia-related BC subtypes adapted to hypoxia through different metabolic pathways.

To identify the potential agents targeting the BC subtypes, we matched the DEG profile of each subtype with the gene profile in CMap database. As a result, 55 compounds referring to 47 mechanisms of action (MoA) were identified targeting subtype 1. These compounds include VEGFR inhibitor motesanib sorafenib, BCR-ABL kinase inhibitor/ABL inhibitor nilotinib and the glutamate receptor antagonist L-701252. Previous research indicated that sorafenib and nilotinib in combination with tamoxifen inhibited growth of tamoxifen-resistant breast cancer cells. The mechanisms of action are complex and both reduced total ER, phosphorylated ER, reduced ligand-independent ER activation due to lowered FOXA1 level, and a switch in the effect of tamoxifen from agonistic to antagonistic *via* reduced AIB1 appears to contribute to growth inhibition (Pedersen et al., 2014). Consistnet with our finding that energy metabolic-related gens were upregulated in subtype 2 breast cancer, compounds involving energy metabolism (such as PI3K inhibitor, mTOR inhibitor, NADH-ubiquinone oxidoreductase inhibitor, and ATP synthase inhibitor) were identified as potential therapeutic agents for subtype 2. In subtype 3, compounds involving regulation of cell cycle and cell apoptosis (including Aurora kinase inhibitor danusertib, CDK inhibitor and caspase inhibitor) were identified as potential therapeutic agents.

However, several limitations in this study should be noted. First, this is a retrospective study, which means that further verification in prospective trials is warranted. Second, the hypoxia-related BC subtypes we identified should be validated externally using different datasets. Finally, the mechanisms underlying our findings have not been clearly elucidated. In other words, experimental studies should be carried out to facilitate our understanding of hypoxia-related gene sets’ functional roles in breast cancer and their clinical application.

## Conclusion

In summary, we identified hypoxia-related BC subtypes based on the enrichment score of 562 hypoxia-associated gene sets. Genes differentially expressed in these BC subtypes correlated with a series of metabolic processes affected by hypoxia. Furthermore, we identified the potential agents targeting the BC subtypes by matching the DEG profile of each subtype with the CMap database. Overall, our study provided a novel classification of BC and identified potential therapeutic agents for each hypoxia-related subtype.

## Data Availability

The original contributions presented in the study are included in the article/Supplementary Material, further inquiries can be directed to the corresponding authors.

## Supplementary Material

Supplementary Figure S1 Hypoxia score of each subtype. **(A)** Subtype 1 displayed a significantly lower Winter score than that of subtype 2 and 3. **(B)** Subtype 1 displayed a significantly lower Ragnum score than that of subtype 2 and 3. **(C)** Subtype 1 displayed a significantly lower Buffa score than that of subtype 2 and 3. For **(A–C)**, *** *p* < 0.001.

Supplementary Figure S2 Composition of clinical and pathological features in each subtypes. **(A)** Composition of Tumor (T) stage in each subtypes. **(B)** Composition of lymph node (N) stage in each subtypes. **(C)** Composition of cancer metastasis (M) stage in each subtypes. **(D)** Composition of molecular subtypes in each subtypes.

Supplementary Figure S3 Survival analysis of hypoxia-related BC subtypes based on METABRICA database. **(A)** Survival analysis of hypoxia-related BC subtypes in BC patients (*n* = 1897). **(B)** Survival analysis of hypoxia-related BC subtypes in luminal A/B BC patients (*n* = 1140). **(C)** Survival analysis of hypoxia-related BC subtypes in Her-2 BC patients (*n* = 219). **(D)** Survival analysis of hypoxia-related BC subtypes in Basal-like patients (*n* = 397). **(E)** Survival analysis of hypoxia-related BC subtypes in BC patients with endocrine therapy (*n* = 1170). **(F)** Survival analysis of hypoxia-related BC subtypes in BC patients with chemotherapy (*n* = 396). For **(A–F)**, *p*-values were determined by log-rank test.
