# Supplementary figures and images for: Breast Cancer Subtypes Based on Hypoxia-Related Gene Sets Identify Potential Therapeutic Agents
Source: Front Mol Biosci. 2022 Jun 29;9:900005. doi: 10.3389/fmolb.2022.900005 (PMC9277110; doi:10.3389/fmolb.2022.900005)

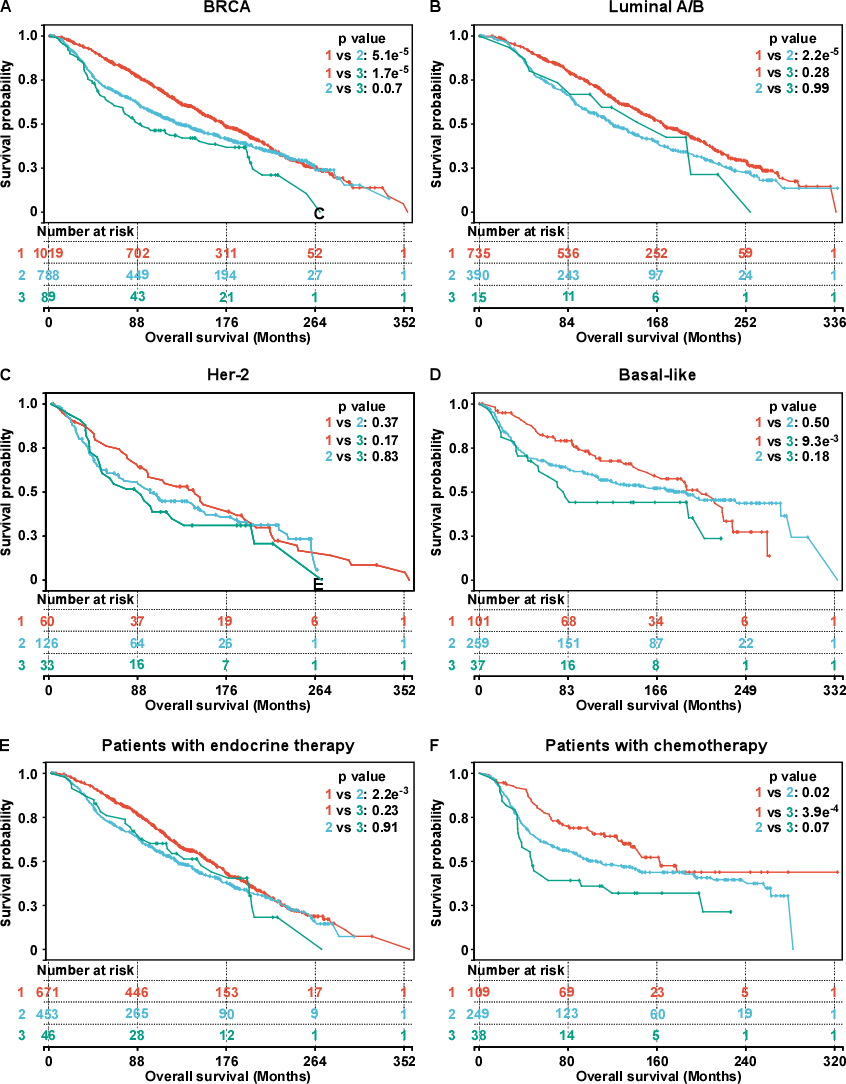

Supplement: Supplementary file 1 [file Image3.TIF]

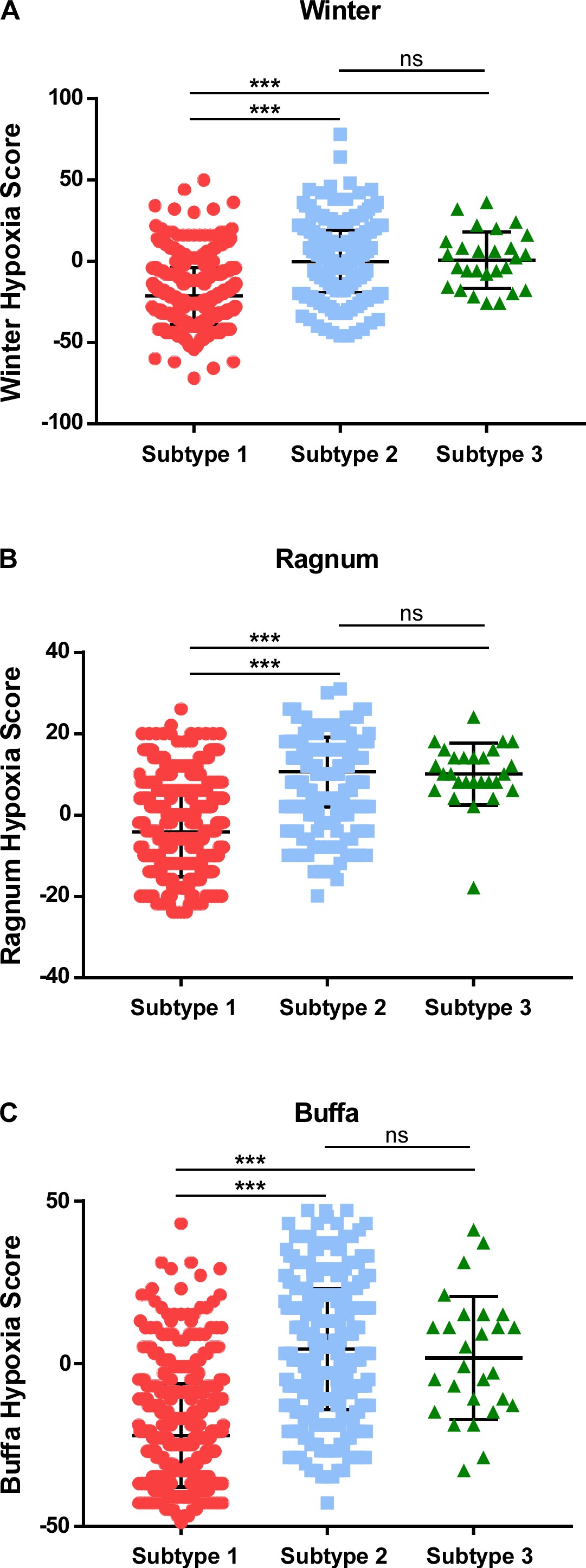

Supplement: Supplementary file 2 [file Image1.JPEG]

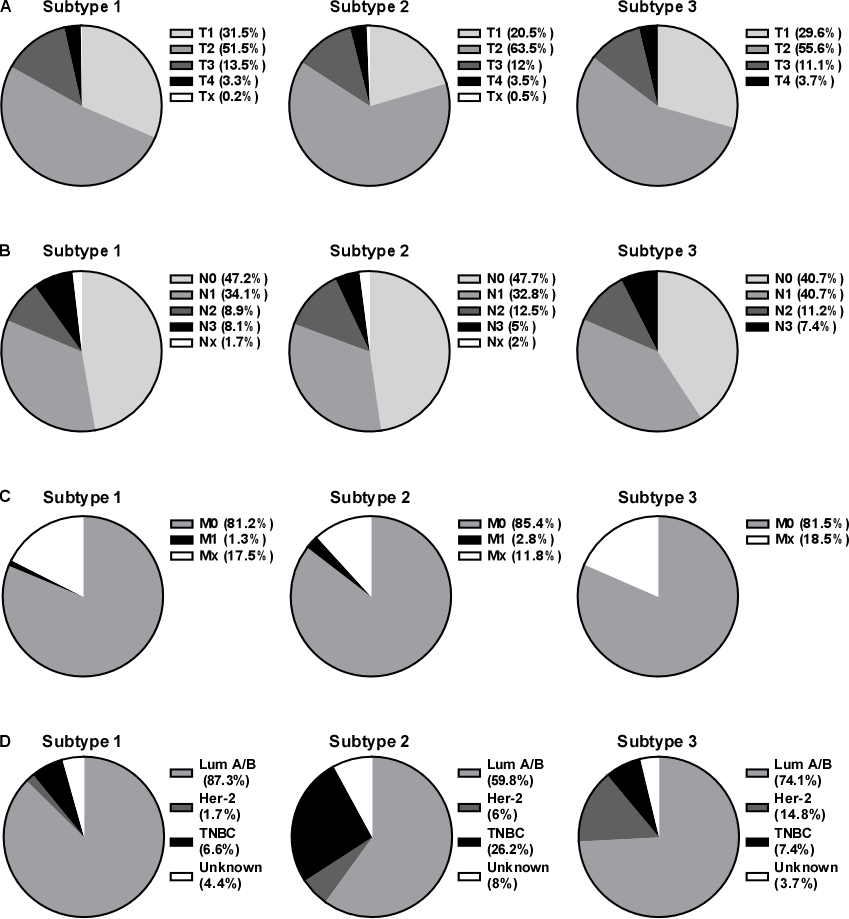

Supplement: Supplementary file 3 [file Image2.TIF]
